# Lasting effects of transcranial direct current stimulation on the inducibility of synaptic plasticity by paired-associative stimulation in humans

**DOI:** 10.1186/s12984-024-01459-x

**Published:** 2024-09-18

**Authors:** Stefan Vestring, Elias Wolf, Johanna Dinkelacker, Sibylle Frase, Carolin Hessling-Zeinen, Shrabon Insan, Maral M. Kumlehn, Bernd Feige, Katharina Domschke, Claus Normann, Lukas Frase

**Affiliations:** 1 Department of Psychiatry and Psychotherapy, Medical Center, University of Freiburg-Faculty of Medicine, University of Freiburg, Hauptstrasse 5, 79104 Freiburg, Germany; 2 Faculty of Science and Engineering, BCN Research Master (C-Tracks), s375081, University of Groningen, Groningen, The Netherlands; 3 Center for Basics in Neuromodulation, Breisacher Str. 64, 79106 Freiburg, Germany; 4 Department of Neurology and Neuroscience, Medical Center, University of Freiburg, Faculty of Medicine, University of Freiburg, Breisacher Str. 64, 79106 Freiburg, Germany; 5 Department of Psychosomatic Medicine and Psychotherapy, Medical Center, University of Freiburg-Faculty of Medicine, University of Freiburg, Hauptstrasse 8, 79104 Freiburg, Germany

**Keywords:** Brain stimulation, Transcranial magnetic stimulation, Cathodal, Anodal, Long-term potentiation

## Abstract

**Background:**

Transcranial direct current stimulation (tDCS) is capable of eliciting changes in cortical neuroplasticity. Increasing duration or repetition of tDCS during the after-effects of a first stimulation has been hypothesized to enhance efficacy. Computational models suggest sequential stimulation patterns with changing polarities to further enhance effects. Lasting tDCS effects on neural plasticity are of great importance for clinical applications.

**Objective:**

The study systematically examined the influence of different tDCS paradigms on long term potentiation (LTP)-like plasticity in humans, focusing on stimulation duration, repetition frequency and sequential combinations of changing polarities as the underlying characteristics.

**Methods:**

Amplitude changes of motor evoked potentials (MEP) were measured in response to paired associative stimulation (PAS) 6 h after application of different tDCS protocols. In total, 36 healthy participants completed the study, randomised into three groups with different stimulation protocols (N = 12 each).

**Results:**

tDCS was able to display lasting modulatory effects on the inducibility of LTP-like plasticity in the human motor cortex 6 h after stimulation. TDCS with the anode on primary motor cortex significantly increased MEP amplitudes following PAS induction. Further analyses highlighted single stimulation block duration to be of higher importance than repetitive protocols for efficacy of effects.

**Conclusions:**

tDCS is capable of inducing lasting changes in the brain’s capability to interact with future stimuli. Especially, effects on the inducibility of LTP-like plasticity might only be detectable with specific tests such as PAS and might otherwise be overlooked. Refined tDCS protocols should focus on higher current and duration of single stimulations instead of implementing complex repetitive schedules.

**Supplementary Information:**

The online version contains supplementary material available at 10.1186/s12984-024-01459-x.

## Background

Transcranial application of weak currents to the human primary motor cortex (e.g. via transcranial direct current stimulation, tDCS) has been repeatedly shown to be capable of eliciting intracortical excitability changes [1–3]. Changes in cortical excitability might lead to a modulation of synaptic plasticity [4, 5] including associative long-term potentiation (LTP) and long-term depression (LTD). Long-term synaptic plasticity represents the basic mechanism for experience-dependent modification of synaptic transmission and has been described in virtually every brain region across species, including humans [6]. While most studies on LTP/LTD are conducted in rodents, LTP-like plasticity in humans can be assessed via amplitude changes of motor evoked potentials (MEP) following paired associative and transcranial magnetic stimulation (PAS) [7]. To assess the interplay of changes in cortical excitability (i.e. excitation-inhibition balance) and associative long-term plasticity, we used tDCS paradigms that were intended to modulate excitation and PAS to read-out their effect on associative LTP [8].

Changes evoked by tDCS evolve during stimulation but persist for prolonged timeframes after stimulation, depending on stimulation polarity, duration and intensity [1, 3, 9, 10]. So far, most studies suggested that excitability modulations induced by tDCS do not exceed 120 min [2, 11]. However, several findings suggest significantly longer tDCS effects depending on differing stimulation protocols: In animal models, repetition of tDCS during the after-effects of a first stimulation session has been shown to enhance efficacy [12]. In humans, repeated tDCS with an interstimulus interval (ISI) of 20 min could elicit prolonged enhancement in motor cortex excitability for approximately 6 h when the anode was placed on the primary motor cortex, whereas temporally contiguous stimulation or longer intervals between stimulation did not induce comparable effects [13]. Some studies even reported polarity-specific EEG differences following repeated tDCS with an interstimulus interval (ISI) of 20 min to last until the next day [14].

Based on recent computational models of neural network dynamics and synaptic plasticity, it has been hypothesized that tDCS triggers neural network remodeling and cell assembly formation [15]. In the same model, repetition of tDCS during after-effects of a prior stimulation (sequential stimulation) as well as reversing stimulation polarity during the ISI (biphasic/polyphasic stimulation) were proposed to enhance the duration and strength of stimulation effects [15]. In animal models, tDCS has been shown to increase survival of synaptic spines and preferential formation of new spines after a combined peripheral stimulation and tDCS [16]. Interestingly, these changes outlasted 24 h and depended on a secondary stimulation paradigm to fully materialize. While most known tDCS effects might rely on functional, short-term neural plasticity and/or a change in excitation-inhibition balance, it remains unclear, how lasting structural neuroplastic changes in humans are best induced and detected.

This study therefore systematically examined different tDCS paradigms regarding their influence on inducibility of LTP-like plasticity in humans, focusing on repetition frequency and sequential combination of changing polarities as potential modifying characteristics. Based on recent findings indicating an important functional and anatomical connection between parietal lobe and motor cortex for coordination of hand movements, we chose a non-typical return electrode positioning over the parietal cortex [17, 18]. We hypothesized that repeated tDCS significantly increases inducibility of LTP-like plasticity when the anode was placed on the primary motor cortex compared to sham stimulation and that reversing polarity of tDCS during the ISI of two (or more; biphasic/polyphasic) tDCS blocks further increases stimulation effects compared to an ISI without stimulation. Secondly, we hypothesized that increasing the number of anodal stimulation blocks significantly enhances LTP-like plasticity, even if the added stimulation duration remains constant. To detect these lasting (structural) changes neural plasticity we applied a paired associative stimulation paradigm several hours later.

## Methods

### Study population

In total, 36 right-handed, healthy participants finished the study protocol (18 females, age range 21–33 years, mean age 24.3 ± 2.8 years). Participants were randomised into three groups with different stimulation protocols (12 participants each; 6 females each; no age difference between groups [*p* = *0.136*]). A narrow age range was chosen to reduce potential age related sources of variance. A thorough screening process including a structured interview [19] was implemented to rule out any relevant mental or somatic disorder or substance use (including smoking and excessive caffeine use > 300 mg/d) as well as any CNS-active medication. Adhering to brain stimulation safety recommendations, subjects with metallic implants or ongoing pregnancy were excluded [20]. The screening process was complemented by self-report questionnaires that ruled out subjective experience of depressive symptoms (Beck Depression Inventory, BDI [21], total score 2.3 ± 2.4; no group difference [*p* = *0.492*]) or excessive daytime sleepiness (Epworth Sleepiness Scale, ESS [22], total score 5.3 ± 3.0; no group difference [*p* = *0.879*]).

Participants were recruited via the internet, official press communications and advertisements of the University Medical Center Freiburg, University of Freiburg, Germany.

### Study design

All experiments were conducted at the Department of Psychiatry and Psychotherapy of the University Medical Hospital Freiburg, Germany.

**Fig. 1:**
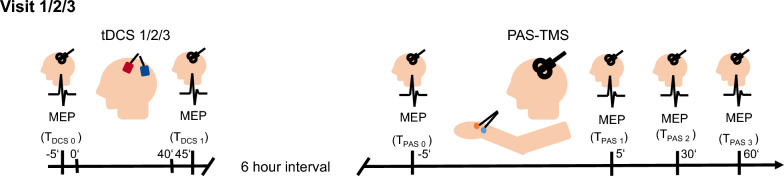
General schedule of one study visit. Following a screening visit, all participants concluded three study visits with tDCS according to the respective experimental protocol during the morning followed by a 6-h break. Afterwards, all participants received paired associative stimulation (PAS) on each visit. Immediately prior to (T_DCS_ 0) and after tDCS (T_DCS_ 1) as well as prior to (T_PAS_ 0) and 5, 30, and 60 min after PAS (T_PAS_ 1–3), motor evoked potentials (MEP) were assessed. Study visits were separated by 1 week. Three differing tDCS protocols (1/2/3) were applied in a balanced, pseudorandomized order, once per visit, for each experiment (A/B/C)

Following a screening visit, all participants concluded three study visits with tDCS according to the respective experimental protocol, followed by motor evoked potentials (MEP) measurements and paired associative stimulation (PAS; see Fig. 1). To avoid confounding metaplastic effects of repeated stimulation, a minimum break of 7 days between each study visit was implemented.

Each of the three study visits began with tDCS according to the scheduled protocol. Following tDCS, participants were instructed to spend the day avoiding any kind of activity involving physical activation (e.g. sportive activities) or daytime napping and to return to the study center in the afternoon. According to recent studies [13], we expected a window of peak effect size concerning inducibility of LTP-like plasticity starting 6 h after the end of tDCS. PAS was therefore induced and measured accordingly. MEPs were recorded prior to and 5, 30, and 60 min after the end of PAS in addition to prior to, and immediately after tDCS.

### Transcranial direct current stimulation (tDCS)

tDCS was delivered by a battery-driven, micro-processor-controlled CE-certified constant current stimulator (neuroConn GmbH, Illmenau, Germany) comprising target electrodes over the right motor cortex and parietal return electrodes (5 × 7 cm each, sponges soaked with 10 ml saline solution) to allow for efficient stimulation of the motor cortex. Electrode placement for the target electrode was guided by using TMS to define the motor cortical representational field of the abductor pollicis brevis muscle (as proposed by Nitsche et al. [1], while the return electrode was placed on an atypical location over the parietal cortex (P3 according to the 10–20 system), following experimental settings capable of inducing long lasting tDCS effects [14] in addition to recent findings indicating an important functional and anatomical connection between parietal lobe and motor cortex for coordination of hand movements [17, 18]. This electrode placement differs from classical electrode montages with the return electrode placed over the contralateral supraorbital area (e.g. [23]). For efficient flow of current and activation of neural structures under the electrodes a minimum distance of around six cm between both electrodes was chosen.

A constant current of 2 mA over each electrode was applied using a fade-in/fade-out design [30 s each] to decrease skin sensations during the beginning and end of the stimulation [24]. A standard sham protocol with 30 s fade-in followed by 30 s fade-out at the beginning and end of each block without active stimulation in between was applied for a duration corresponding to the respective anodal stimulation setting [25]. For each participant, one of the following predefined orders of experimental protocols was chosen in a pseudorandomized and balanced order based on study entry to prevent sequential effects: 1–2–3, 3–1–2 or 2–3–1. Polarity setting 1 was designed to induce strong effects by placing the anode on the primary motor cortex; setting 2 was focused on optimizing effects of tDCS with the cathode on the primary motor cortex; while setting 3 represented the sham condition for each experiment (nomenclature according to [26]).

For experiment B and C, more than one stimulation block was applied (biphasic/polyphasic). To stay within safety recommendations and to keep the conditions as comparable as possible, the total stimulation duration was kept constant (e.g. 20 min of tDCS with the anode on the primary motor cortex).

**Fig. 2:**
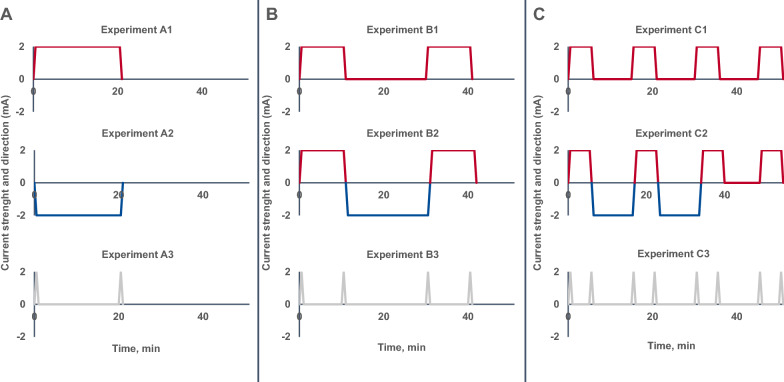
Overview of applied tDCS protocols.** A** Stimulation protocols for experiment A. Participants either received 20 min. of 2 mA tDCS with the anode on the primary motor cortex (A1), 20 min. of 2 mA tDCS with the cathode on the primary motor cortex (A2) or sham stimulation (A3).** B** Stimulation protocols for experiment B. Participants either received two blocks of 10 min. of 2 mA tDCS with the anode on the primary motor cortex (interstimulus interval [ISI] of 20 min.; B1), two blocks of 10 min. of 2 mA tDCS with the anode on the primary motor cortex, with 20 min. of 2 mA tDCS with reversed polarity in between (B2) or sham stimulation (B3).** C** Stimulation protocols for experiment C. Participants either received four blocks of 5 min. of 2 mA tDCS with the anode on the primary motor cortex (interstimulus interval [ISI] of 10 min.; C1), four blocks of 10 min. of 2 mA tDCS with the anode on the primary motor cortex with two blocks of 10 min. of 2 mA tDCS with reversed polarity in between (C2), or sham stimulation (C3)

*Experiment A* implemented one singular block of stimulation for a duration of 20 min to allow for the longest constant stimulation time at the chosen intensity with regards to standard safety criteria [20]. Polarity settings 1–3 therefore comprised 20 min of tDCS with either the anode (A1) or cathode (A2) on the primary motor cortex, or sham tDCS (A3; see Fig. 2A).

*Experiment B* examined the effect of two sequential 10 min stimulation blocks with the anode on the primary motor cortex, with either a 20 min interstimulus interval (ISI; B1) or 20 min of tDCS with reversed polarity in between (B2). Two respective blocks of sham stimulation with an ISI of 20 min served as the sham condition (B3; see Fig. 2B).

*Experiment C* further prolonged the sequential stimulation pattern, with C1 including four blocks of 5 min of tDCS with the anode on the primary motor cortex, with three ten minutes ISI in between. For experimental sequence C2, two of the three ISI were replaced by 10 min of tDCS with reversed polarity each. To adhere to standard safety parameters, a total stimulation time of 40 min could not be exceeded. Therefore, only two of the three ten minutes ISIs were exchanged (see Fig. 2C).

### Paired associative stimulation—transcranial magnetic stimulation (PAS)

TMS was applied by standard criteria using a figure-of-eight coil with an outer diameter of 90 mm connected to a Magstim 200 stimulator (The Magstim Company, Whitland, UK). Optimal coil placement was defined as tangentially to the skull over the right primary motor cortex (M1) with the handle pointing in a posterior direction, at a lateral angle of 45° regarding the midline. To identify the optimal coil position for eliciting MEPs of maximal amplitude of the left abductor pollicis brevis (APB) muscle (‘hotspot’), the coil was moved over M1 while administering 0.25 Hz stimuli (suprathreshold intensity). The identified coil position was then recorded using a stereotaxic, optically tracked navigation system, consisting of a camera (Polaris Vicra P6, NDI, Waterloo, ON, Canada), custom-made software (Visor2, eemagine GmbH, Berlin, Germany), and passive sphere markers [27], and kept constant throughout measurements.

Resting motor threshold (RMT) was determined according to standard criteria [28], with stimulation intensity for MEP measurements adjusted to elicit MEPs with peak-to-peak amplitudes of on average 600–1400 μV (SI 1 mV). At each measuring point, twenty TMS pulses were administered at a frequency of 0.1 Hz and the corresponding peak-to-peak amplitudes of these pulses were averaged using Signal Software (CED, UK). For optimal MEP recordings, participants were instructed to relax the targeted left APB muscle during all measurements, which was monitored visually via a concurrent electromyogram (EMG). MEPs were recorded using silver/silver chloride electrodes (AMBU, Ballerup, Denmark) in a belly-tendon montage. Signals were band-pass filtered (20–2000 Hz), amplified using an Ekida DC universal amplifier (EKIDA GmbH, Helmstadt, Germany), digitized at a 5 kHz sampling rate using a MICRO1401mkII data acquisition unit (CED), and stored on a computer for offline analysis. MEPs with preceding muscle activity were excluded from analysis. MEPs were normalized by diving each post-PAS MEP with the corresponding pre-PAS MEP.

The chosen PAS protocol closely follows standard procedures [7]. In summary, the protocol comprised 200 pairs of peripheral and cortical stimuli, given at a frequency of 0.25 Hz (total duration ~ 13 min). The peripheral pulse was delivered to the median nerve of the left wrist at an intensity of 300% of the sensory perceptual threshold by a Digitimer DS7 electrical stimulator (Digitimer, Welwyn Garden City, Hertfortshire, UK) as constant current square wave pulses with a duration of 1000 μs.

The interstimulus interval (ISI) between the peripheral and cortical stimulation was set to 25 ms. Participants were instructed to direct their attention to the stimulated hand and count rarely occurring (4 stimuli in total), randomly intermittent, electrical stimuli to the thumb of the stimulated hand (200% perceptual threshold, cathode proximal, constant current square wave pulses, duration 200 μs), that were administered during the ISI to decrease influences of differing attention levels [29–31]. These stimuli were administered through an additional electrode placed distal to the peripheral electrode on the thumb.

### Statistical analyses

MEP mean amplitudes were considered as primary outcome parameters. To test for MEP amplitude differences, repeated-measures analyses of variance (ANOVA) with the within-subject factors condition (tDCS protocols) and timepoint (T_DCS_ 0–1, T_PAS_ 0–3) were conducted for each experiment. Simple two-tailed t-tests were conducted post-hoc to test for differences between specific MEPs. In addition, one sample t-test comparing the sample mean against a hypothetical mean of 1 (due to normalization) were conducted (Fig. 7).

Descriptive values are given as means and standard deviations. For the estimation of effect sizes, partial ETA squared values were calculated (low: < 0.06; medium: ≥ 0.06 and < 0.14; large: ≥ 0.14). The level of significance was set at *p* < *0.05* (two-tailed). In cases of violations of sphericity, the Greenhouse–Geisser adjustment was applied. For subgroup analysis PAS response was defined as reaching a normalized (to baseline) MEP amplitude greater than one at T_PAS_ 2 in the sham condition. All analyses were conducted with the statistical software IBM SPSS Statistics (Version 29).

## Results

### No short-term and long-term MEP amplitude differences after tDCS

To control for immediate and delayed excitability increasing effects of the different tDCS protocols, we compared MEP amplitudes prior to and after tDCS (T_DCS_ 0–1). MEP amplitudes did not differ in any of the experiments (experiment 1: timepoint: F = 0.7, *p* = *0.792*, pETA^2^ = 0.007; condition: F = 0.6, *p* = 0.5*76*, pETA^2^ = 0.115; interaction timepoint x condition: F = 0.6, *p* = 0.5*76*, pETA^2^ = 0.115; experiment 2: timepoint: F = 1.1, *p* = *0.308*, pETA^2^ = 0.094; condition: F = 0.07, *p* = 0.9*32*, pETA^2^ = 0.014; interaction timepoint × condition: F = 0.07, *p* = 0.9*32*, pETA^2^ = 0.014; experiment 3: timepoint: F = 1.2, *p* = *0.306*, pETA^2^ = 0.095; condition: F = 1.3, *p* = 0.3*12*, pETA^2^ = 0.208; interaction timepoint × condition: F = 1.3, *p* = 0.3*12*, pETA^2^ = 0.208).

Moreover, MEP amplitudes post tDCS and pre PAS did not differ in any condition (experiment 1: timepoint: F = 1.186, *p* = *0.2840*; condition: F = 2.927, *p* = 0.0676; interaction timepoint × condition: F = 1.238, *p* = 0.3030; experiment 2: timepoint: F = 3.180, *p* = *0.6102*; condition: F = 2.229, *p* = 0.1236; interaction timepoint × condition: F = 0.5015, *p* = 0.6102; experiment 3: timepoint: F = 0.007446, *p* = *0.9318*; condition: F = 0.09192, *p* = 0.9*124*; interaction timepoint × condition: F = 0.5729, *p* = 0.5694).

### Induction of long-term potentiation-like plasticity by paired associative stimulation

**Fig. 3:**
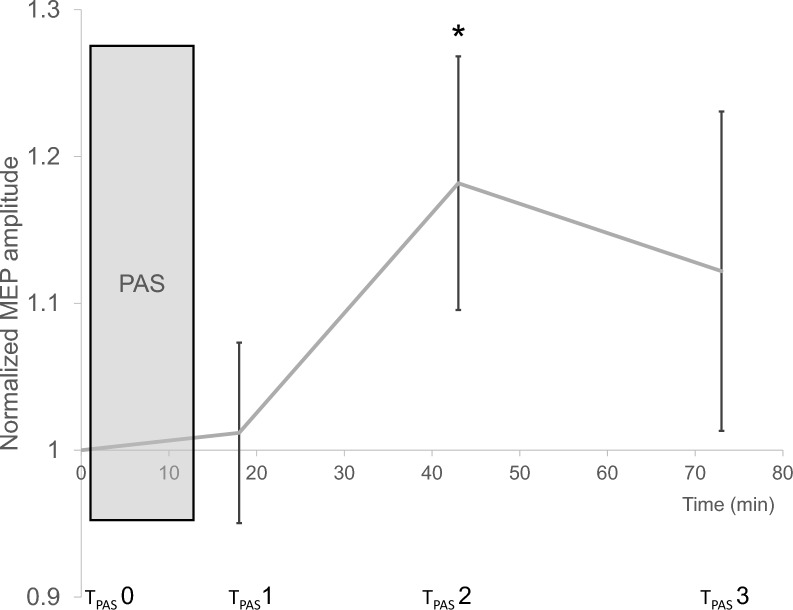
Induction of long-term potentiation by PAS. Effects of paired associative stimulation (PAS) on motor evoked potentials (MEP) amplitudes were measured at three timepoints following PAS induction (T_PAS_ 1–3) and compared to a baseline measurement immediately prior to PAS (T_PAS_ 0). T_PAS_ 2 displayed significantly increased MEP amplitudes compared to T_PAS_ 0. Normalized MEP amplitudes, averaged across all experiments. Means ± SEM

Effects of PAS on MEP amplitude were measured at three timepoints at 5, 30 and 60 min following PAS induction (T_PAS_ 1–3) and compared to a baseline measurement immediately prior to PAS (T_PAS_ 0; see Fig. 3). To assert the general feasibility of the chosen PAS paradigm, averaged data across all sham conditions (A3, B3, C3; N = 36) was analyzed. Focusing on the timepoint displaying the largest effect on LTP-like plasticity, which was expected around 30 min after conclusion of PAS (T_PAS_ 2), MEP amplitudes significantly differed from baseline with PAS inducing an increase of 0.18 µV (F = 4.4, *p* = *0.042*, pETA^2^ = 0.112).

### Lasting modulatory effects of single block tDCS (experiment A)

One singular block of tDCS for a duration of 20 min led to a polarity-specific modulation of inducibility of LTP-like plasticity by PAS 6 h later (for stimulation protocol see Fig. 2A). Specifically, a significant interaction between timepoint of MEP measurement and condition of stimulation protocol could be detected (F = 2.9, *p* = 0.0*13*, pETA^2^ = 0.211; timepoint: F = 1.5, *p* = *0.233*, pETA^2^ = 0.120; condition: F = 0.9, *p* = 0.4*36*, pETA^2^ = 0.073).

**Fig. 4:**
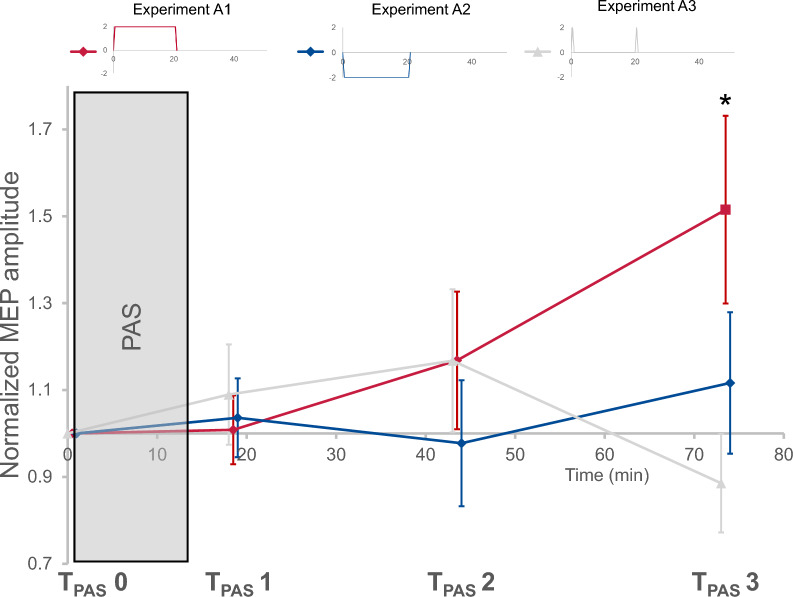
Lasting modulatory effects of single block transcranial direct current stimulation (tDCS). Main effects of tDCS on inducibility of LTP-like plasticity. Effects of paired associative stimulation (PAS) on motor evoked potential (MEP) amplitudes were measured at three timepoints following PAS induction (T_PAS_ 1–3) and compared to a baseline measurement immediately prior to PAS (T_PAS_ 0). A significant interaction between timepoint of MEP measurement and stimulation protocol could be detected. Post-hoc testing revealed T_PAS_ 3 to display a significantly higher MEP amplitude compared to T_PAS_ 0 following tDCS with the anode on the primary motor cortex (experiment A1, indicated by *). In addition, at T_PAS_ 3, anodal tDCS led to significantly higher MEP amplitudes compared to sham (not marked). Means ± SEM

Post-hoc testing revealed that tDCS with the anode on the primary motor cortex was the major factor of the significant interaction, with T_PAS_ 3 displaying a significantly higher MEP amplitude compared to T_PAS_ 0 (A1, F = 5.7, *p* = 0.0*36*, pETA^2^ = 0.341). In addition, tDCS with the anode on the primary motor cortex led to significantly higher MEP amplitudes at T_PAS_ 3 compared to sham (F = 6.1, *p* = *0.032*, pETA^2^ = 0.355). Interestingly, while the influence of prior tDCS with the anode on the primary motor cortex led to a later rise in MEP amplitudes following PAS than expected and detected after sham stimulation, participants displayed no increase in MEP amplitude following tDCS when the cathode was placed on the primary motor cortex (A2), which suggests an induced suppression of the expected PAS effects (see Fig. 4). For individual data please refer to Fig. S1 (supplements).

### No lasting modulatory effects of sequential tDCS (experiment B)

Sequential blocks of tDCS did not lead to a significant protocol-specific modulation of inducibility of LTP-like plasticity by PAS 6 h later (for stimulation protocol see Fig. 2B; timepoint: F = 2.8, *p* = 0.0*54*, pETA^2^ = 0.204; condition: F = 2.8 *p* = 0.0*85*, pETA^2^ = 0.201; interaction timepoint x condition, F = 1.7, *p* = 0.1*97*, pETA^2^ = 0.133).

**Fig. 5:**
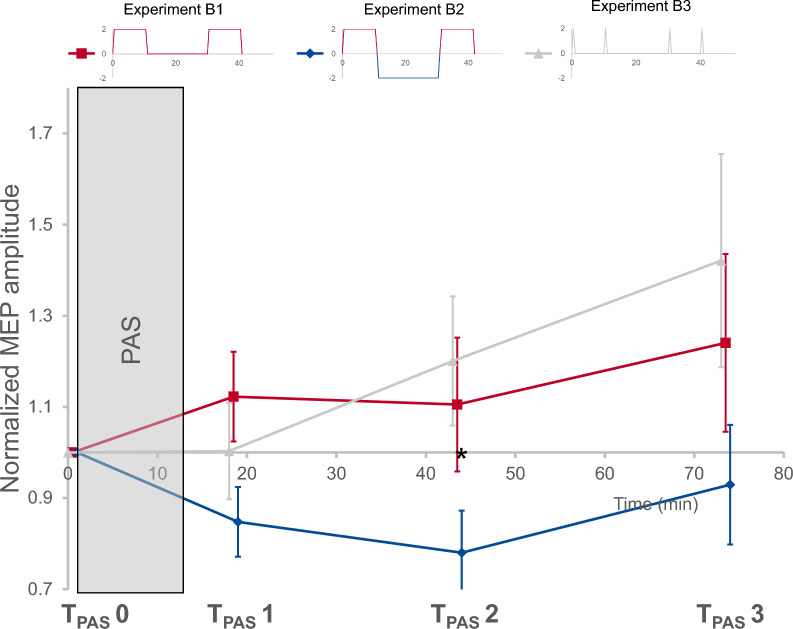
No lasting modulatory effects of sequential transcranial direct current stimulation (tDCS). Main effects of tDCS on inducibility of LTP-like plasticity. Effects of paired associative stimulation (PAS) on motor evoked potential (MEP) amplitudes were measured at three timepoints following PAS induction (T_PAS_ 1–3) and compared to a baseline measurement immediately prior to PAS (T_PAS_ 0). No main effects were found. Exploratory post-hoc testing indicated biphasic sequential tDCS with direction changes of the electrical field (experiment B2) to induce significantly lower MEP amplitudes at T_PAS_ 2 compared to T_PAS_ 0 (indicated by *). In addition, at T_PAS_ 2, biphasic sequential tDCS led to significantly lower MEP amplitudes compared to monophasic tDCS (not marked). Means ± SEM

Exploratory post-hoc testing indicated that the most prominent differences were between T_PAS_ 2 and T_PAS_ 3 where all conditions displayed an increase in MEP amplitudes (F = 5.3, *p* = 0.0*42*, pETA^2^ = 0.324). In addition, biphasic sequential tDCS displayed significantly lower MEP amplitudes at T_PAS_ 2 compared to T_PAS_ 0 (B2, F = 5.7, *p* = 0.0*37*, pETA^2^ = 0.340) and compared to monophasic sequential tDCS at T_PAS_ 2 (B1, F = 6.9, *p* = *0.023*, pETA^2^ = 0.387; see Fig. 5). For individual data please refer to Fig. S1 (supplements).

### No lasting modulatory effects of sequential tDCS at higher frequencies (experiment C)

**Fig. 6:**
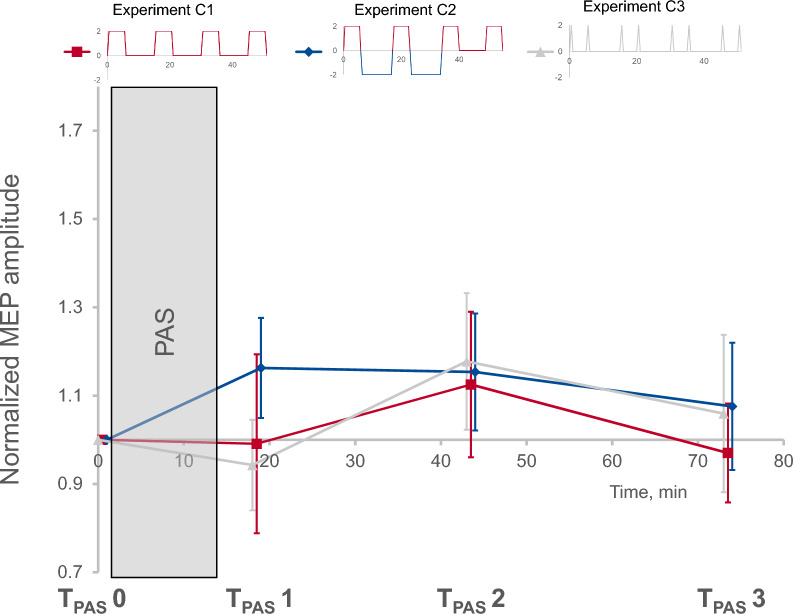
No lasting modulatory effects of sequential transcranial direct current stimulation (tDCS) at higher frequencies. Main effects of tDCS on inducibility of LTP-like plasticity. Effects of paired associative stimulation (PAS) on motor evoked potential (MEP) amplitudes were measured at three timepoints following PAS induction (T_PAS_ 1–3) and compared to a baseline measurement immediately prior to PAS (T_PAS_ 0). No significant modulation of inducibility of LTP-like plasticity could be detected. Means ± SEM

Sequential blocks of tDCS with higher frequency but reduced duration of singular stimulation blocks (see Fig. 2C) failed to induce a significant modulation of inducibility of LTP-like plasticity by PAS 6 h later (timepoint: F = 0.8, *p* = *0.488*, pETA^2^ = 0.070; condition: F = 0.3, *p* = *0.745*, pETA^2^ = 0.026; interaction timepoint × condition: F = 0.4, *p* = *0.879*, pETA^2^ = 0.035; see Fig. 6). For individual data please refer to Fig. S1 (supplements).

## Discussion

### General duration and characterization of tDCS effects in humans

A first major conclusion is that tDCS was able to produce lasting modulatory effects on the inducibility of LTP-like plasticity in the human motor cortex. Direct effects of tDCS on excitability were probably prevented by the atypical electrode positioning. Even in the absence of immediate effects on the MEP amplitudes, the chosen tDCS protocols did show polarity- and frequency-specific effects on the inducibility of neuroplastic changes by PAS 6 h later. These results indicate that maintained tDCS effects other than direct excitability changes modulate associative long-term plasticity induction, even after 6 h. This could be due to complex metaplastic changes (i.e. changes of excitation-inhibition balance), which might be of decisive importance for the underlying long-term plasticity processes frequently reported effective in motor rehabilitation [32, 33].

These findings add to the data on long-term tDCS effects and demonstrate, for the first time, clear changes in the response to plasticity-modulating interventions as late as 6 h after tDCS.

Specifically, 20 min of sustained tDCS with the anode on the primary motor cortex (experiment A1) led to a significant long-term boost of inducibility of LTP-like plasticity by PAS. While PAS regularly led to an increase in MEP amplitudes with a maximum after around 30 min following sham tDCS, sustained 20 min tDCS with the anode on the primary motor cortex led to a slower increase in MEP amplitudes reaching a maximum at around 60 min past PAS. At this timepoint, MEP amplitudes were significantly higher compared to sham stimulation and significantly higher than baseline values. As no further MEPs were measured, it remains unclear how long the induced MEP increase would have been sustained.

### Repetitive tDCS blocks are not superior to sufficiently powered single stimulations

**Fig. 7:**
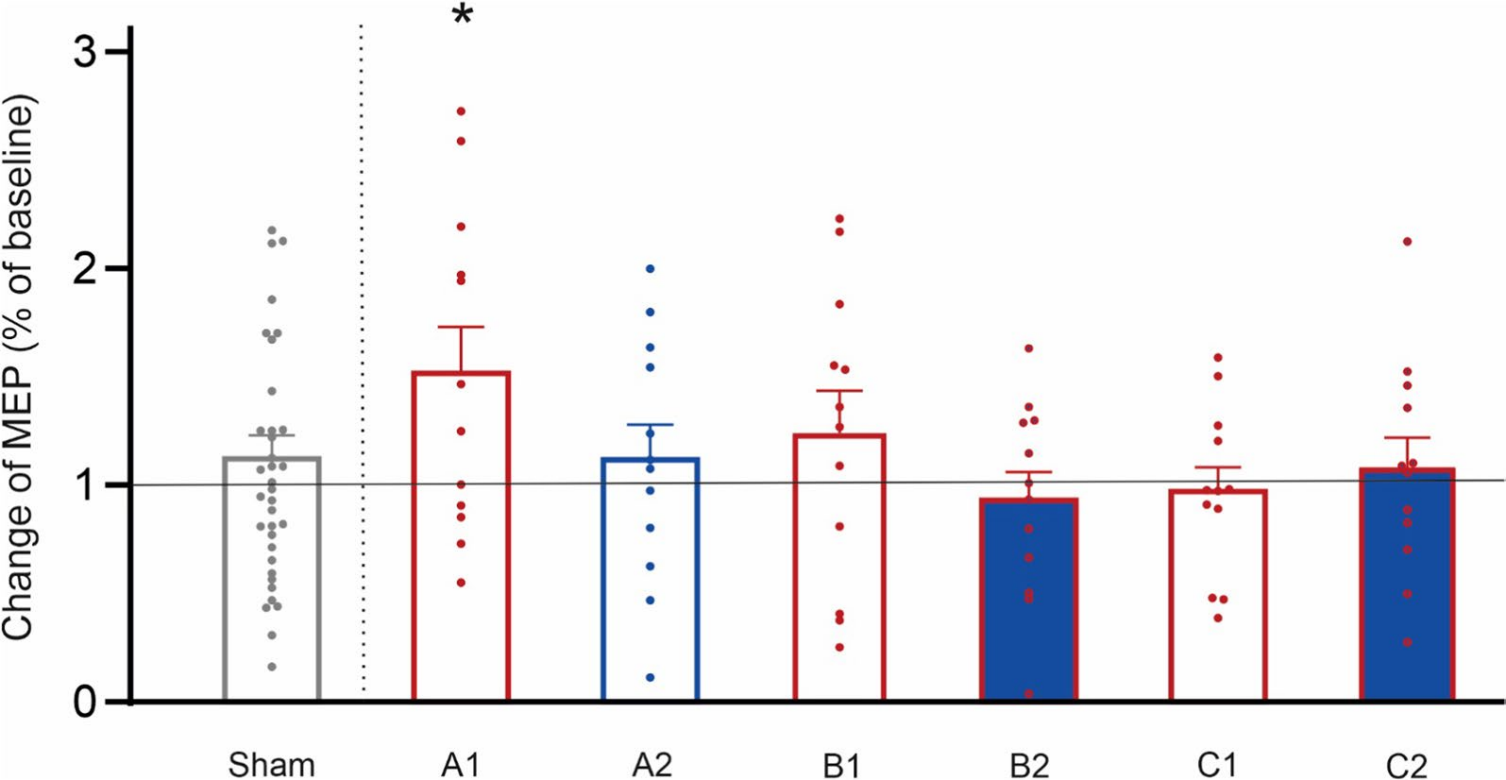
Overview of normalized motor evoked potentials (MEP) amplitudes at T_PAS_ 3. Effects of paired associative stimulation (PAS) on motor evoked potential (MEP) amplitudes 60 min. after induction (T_PAS_ 3) indicated a clear increase only following transcranial direct current stimulation with the anode on the primary motor cortex (tDCS; experiment A1), with all other tDCS protocols showing no difference to sham (averaged across all experiments for this visualization). One sample t-test comparing the sample mean against a hypothetical mean of 1 (due to normalization): A1: p = 0.0362*, A2: p = 0.2432, B1: p = 0.7951, B2: p = 0.4900, C1: p = 0.6004, C2: p = 0.6099, Sham: p = 0.2698. Means ± SEM

Despite our initial hypothesis and modelling data hinting at positive effects [15], repetitive tDCS protocols did not increase tDCS efficacy in this study (experiments B1/C1). It appears that the duration of singular stimulation blocks is highly important for the longevity of effects and multiple shorter stimulation blocks do not add up to reach the same effect size. Instead, shorter stimulation blocks as typically applied in sequential settings appear underpowered to induce lasting effects. Comparing normalized amplitudes at T_PAS_ 3 (60 min after stimulation) across settings, only 20 min and 2 × 10 min monophasic tDCS with the anode on the primary motor cortex showed increased amplitudes, with only 20 min tDCS with the anode on the primary motor cortex displaying changes superior to averaged sham response across all conditions (see Fig. 7). A different interpretation could be, that tDCS protocols with more than one stimulation block inhibit later inducibility of LTP-like plasticity (e.g. by inducing stronger short-term effects with inhibited afterphases). However, we did not detect stronger short-term effects for those protocol in our (limited) MEP data.

The findings are in line with animal model data hinting at higher stimulation intensities needed to affect neural circuits than historically expected [34]. The authors recommend 4–6 mA instead of the state-of-the-art usage of 1–2 mA (as applied in this study) [34]. However, experimental data in humans did not demonstrate a clear correlation between increasing current intensity and effect sizes [2, 35]. For cathodal tDCS, the relationship between stimulation intensity, repetition frequency, and effect size and direction appears more complex with some intensities leading to diminished effects, others to anodal-tDCS-like enhancement of neuroplasticity [2, 15]. To date, general recommendations for optimal stimulation range between 1 and 3 mA [35, 36].

### Differential effects of tDCS with direction changes of the electrical field (anodal/cathodal)

In experiment A2, PAS was not able to induce LTP-like effects after 20 min of tDCS with the cathode on the primary motor cortex earlier in the day. Instead, in experiment B2, the biphasic setting of two-times 10 min of tDCS with the anode on the primary motor cortex with 20 min of reversed polarity tDCS during the ISI showed a significant reduction of MEP amplitudes at T_PAS_ 2 compared to T_PAS_ 0, and compared to two-times monophasic 10 min tDCS without stimulation during the ISI. TDCS with the cathode on the primary motor cortex appears to have a diminishing effect on inducibility of LTP-like plasticity that might be enhanced by carefully combining phases of changing polarity. In contrast to our initial hypothesis, the polarity of the longest single tDCS block might determine the direction of the effect more than the order of stimulations. We initially designed the protocol to increase the effect size of two tDCS blocks with the anode on the primary motor cortex by adding tDCS in reverse polarity to the ISI. However, the resulting paradigm can be better interpreted as a tDCS of 20 min duration with the cathode on the primary motor cortex, with 10 min of reversed polarity tDCS beforehand acting as a preconditioning effect (priming; [37]). It is to note, though, that all interpretations of tDCS effects with the cathode on the primary motor cortex in this dataset rely on exploratory data and lack robust statistical support.

### Limitations

The study was conceptualized to further understand ongoing tDCS effects that might influence the brains capability to interact with stimuli long after conclusion of stimulation (e.g. [14]). The electrode placement, mainly the placement of the ‘return’ electrode on the parietal cortex was chosen based on recent findings indicating the importance of the parietal cortex specifically for hand movements [17, 18]. In addition, this electrode placement reduced the direct effects of tDCS on excitability and MEP amplitudes, thereby improving the measurability of long-term plasticity induced by PAS. However, the resulting electric field is different compared to the classical electrode placement (over M1 and the contralateral supraorbital area) and may not necessarily be comparable to studies using the standard montage.

In addition, the chosen electrode placement might have reduced the expected efficacy of the stimulation by resulting in a distance between electrodes slightly smaller compared to the standard electrode montage [38]. In general, choosing high definition tDCS approaches might be useful to further clarify the results [39].

As can be seen in Figs. 4 and 5, MEP changes following sham stimulation depicted a slightly different trajectory across experiments and only clearly displayed the expected changes when combined across experiments (N = 36, Fig. 3). To our understanding, these differences are most likely due to random variations in stimulation response and pronounced by a limited sample size [40]. This might add to overall variance and diminish the explanatory power of the results. However, exploratory analysis of subgroups with a clear PAS response following sham supported the main analysis (lower significance levels due to smaller sample size of N = 7–8, supplements Fig. S2).

The current study aimed at understanding long-term effects of tDCS on neuroplasticity given the heterogeneity of results in previous clinical studies.

As the study was conducted in healthy participants, results might not translate to populations with potentially disturbed levels of neuroplasticity as in major depressive disorder (MDD). In addition, the study examined neuroplastic effects in the motor cortex, while from a clinical perspective other areas, e.g. prefrontal brain regions, might be of higher importance. However, there is growing support on the transferability of neurostimulation effects between brain regions [41].

## Conclusions

The current study shows tDCS to be capable of inducing long term plasticity-inducing effects several hours after stimulation. This effect duration might be frequently overlooked in regular study designs because detection relies on specific interventions to discriminate underlying effects on inducibility of neural plasticity [5]. PAS has the potential to provide a tool for detecting these long-term, possibly structural, tDCS effects. In addition, the study adds to the understanding of the interaction between repetition patterns and stimulation intensity in designing optimal tDCS protocols [15].

Besides promising results in initial clinical trials [42, 43], tDCS recently failed to demonstrate efficacy in augmenting standard treatment for MDD patients [44, 45]. However, plasticity processes are not immediate, and antidepressant treatment response requires long-lasting restorative effects [46]. The underlying expectation of therapeutic effects of time-limited interventions by tDCS implies the existence of longer lasting effects than commonly conceptualized. In addition, the lacking clinical effect appears to be inconsistent with a growing body of data on plasticity-modulating tDCS effects (e.g. [5]).

As a conclusion, there is a pressing need for refined tDCS protocols [47]. In analogy to optimized rTMS protocols using pulsed protocols [48] and predictions from modelling studies [15], it has been expected that spaced, sequential tDCS would be more effective. Regarding short-term effects on MEP amplitudes and long-term effects on synaptic plasticity, we could not confirm this assumption. Therefore, further research on optimized tDCS protocols is necessary [47, 49].

## Supplementary Information

Supplementary Material 1: Fig. S1: Systematic presentation of all experimental series including individual data. Means ± SEM

Supplementary Material 2: Fig. S2: Differential effects of paired associative stimulationon motor evoked potentialamplitudes in a subgroup of defined PAS responders depending on prior tDCS. PAS responders were defined by reaching a normalizedMEP amplitude greater than one at T_PAS_ 2 in the sham condition.. Means ± SEM.

## Data Availability

The datasets generated and/or analysed during the current study are not publicly available due to German data protection laws but are available in accordance with these from the corresponding author on reasonable request.

## Abbreviations

tDCS: Transcranial direct current stimulation
ESS: Epworth Sleepiness Scale
BDI: Beck Depression Inventory
PAS: Paired associative stimulation
MEP: Motor evoked potentials
TMS: Transcranial magnetic stimulation
LTP: Long-term potentiation
LTD: Long-term depression
ISI: Interstimulus interval
APB: Abductor pollicis brevis
RMT: Resting motor threshold
CNS: Central nervous system
MDD: Major depressive disorder
