# Supplementary figures and images for: Lasting effects of transcranial direct current stimulation on the inducibility of synaptic plasticity by paired-associative stimulation in humans
Source: J Neuroeng Rehabil. 2024 Sep 18;21:162. doi: 10.1186/s12984-024-01459-x (PMC11409632; doi:10.1186/s12984-024-01459-x)

Condition 1

Experiment A

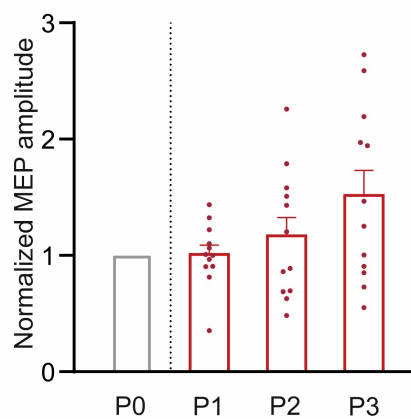

Experiment B

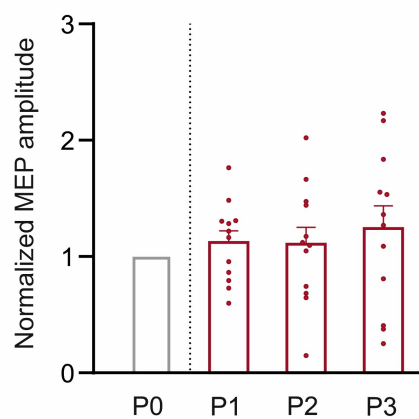

Experiment C

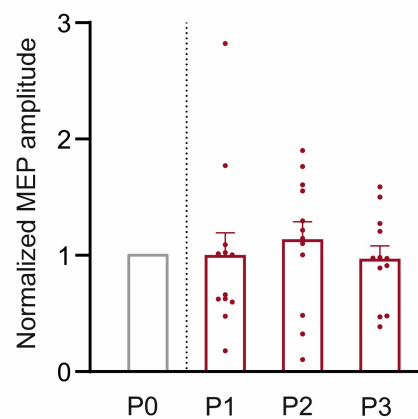

Condition 2

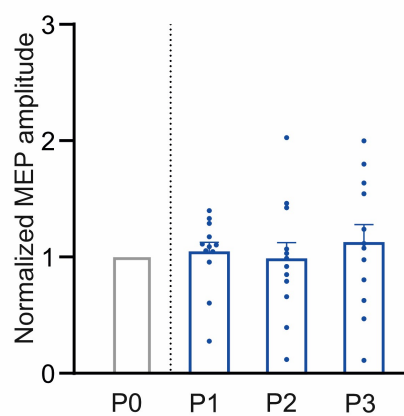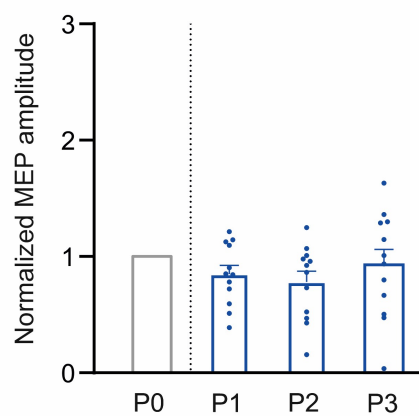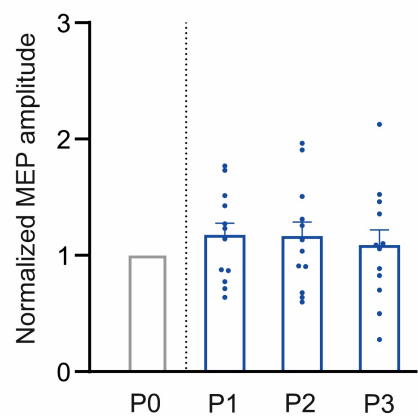

Condition 3

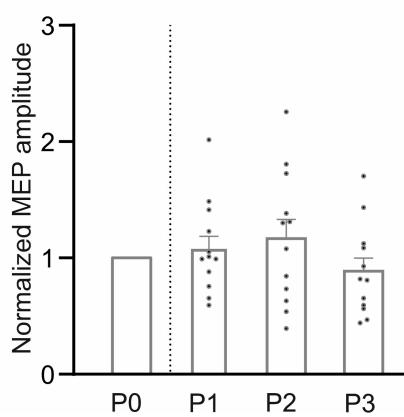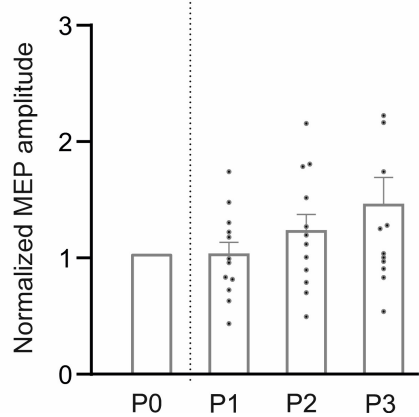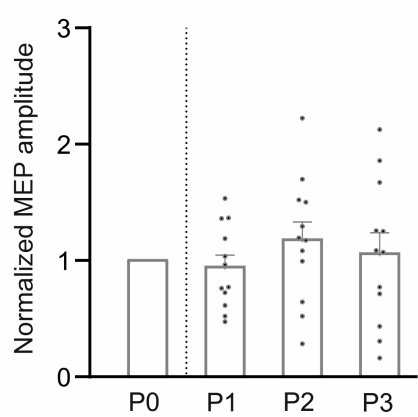

Supplement: Supplementary file 1 — Supplementary Material 1: Fig. S1: Systematic presentation of all experimental series including individual data. Means ± SEM [file 12984_2024_1459_MOESM1_ESM.pdf]
