# Supplementary material for: Lasting effects of transcranial direct current stimulation on the inducibility of synaptic plasticity by paired-associative stimulation in humans
Source: J Neuroeng Rehabil. 2024 Sep 18;21:162. doi: 10.1186/s12984-024-01459-x (PMC11409632; doi:10.1186/s12984-024-01459-x)
Supplement: Supplementary file 2 — Supplementary Material 2: Fig. S2: Differential effects of paired associative stimulationon motor evoked potentialamplitudes in a subgroup of defined PAS responders depending on prior tDCS. PAS responders were defined by reaching a normalizedMEP amplitude greater than one at TPAS 2 in the sham condition.. Means ± SEM. [file 12984_2024_1459_MOESM2_ESM.pptx]

## Slide 1
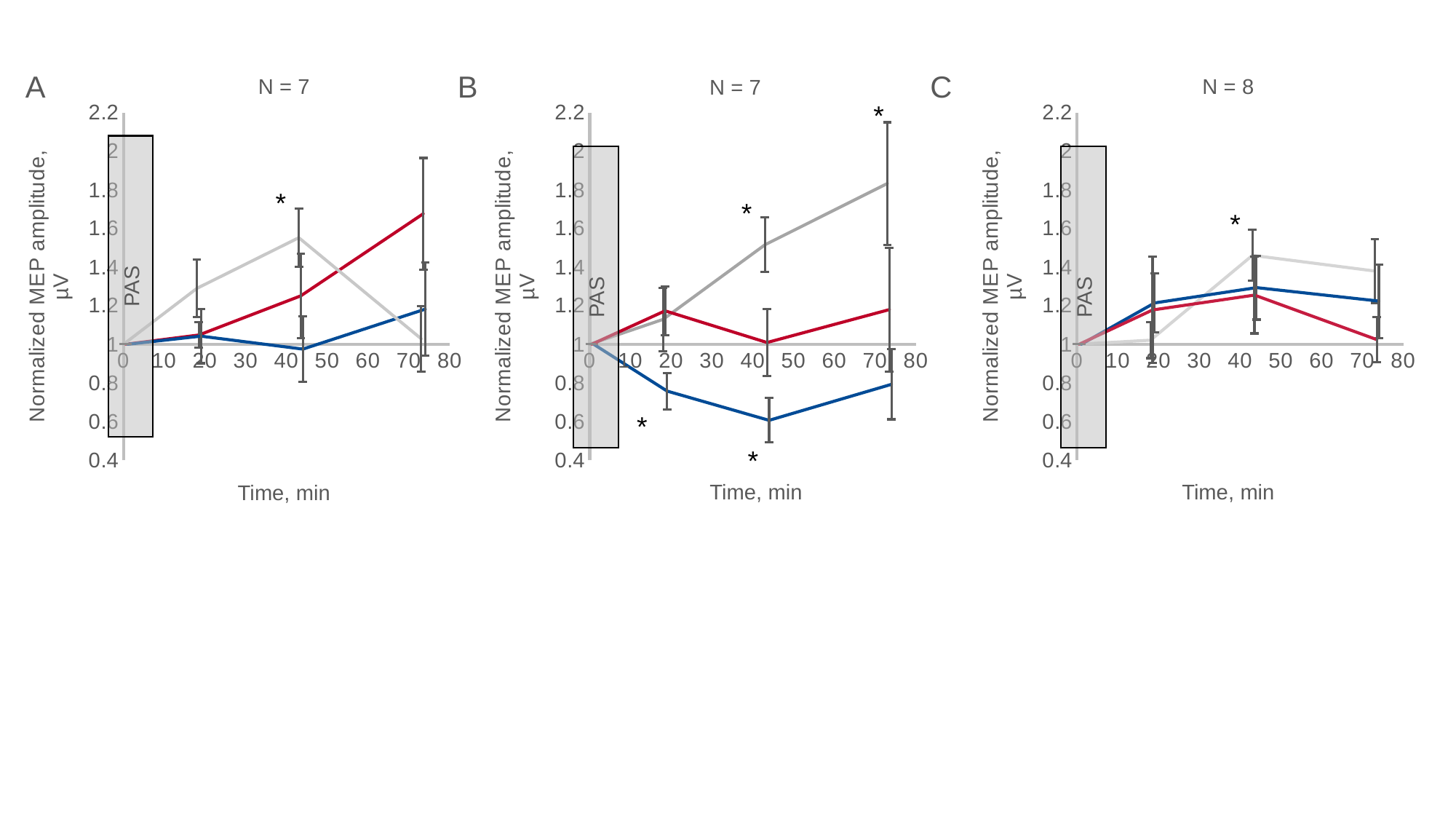

A
B
C
N = 8
N = 7
N = 7
### Chart
| Category | | | |
|---|---|---|---|
### Chart
| Category | | | |
|---|---|---|---|*
### Chart
| Category | | | |
|---|---|---|---|PAS
PAS
PAS
*
*
*
*
*
Time, min
Time, min
Time, min
